# Knowledge and beliefs about the use/abuse of oral contraceptive pills among males: A mixed-method explanatory sequential study in community pharmacy settings

**DOI:** 10.1371/journal.pone.0251302

**Published:** 2021-05-07

**Authors:** Muna Barakat, Raja’a Al-Qudah, Amal Akour, Mona Abu-Asal, Samar Thiab, Yahya H. Dallal Bashi

**Affiliations:** 1 Faculty of Pharmacy, Applied Science Private University, Amman, Jordan; 2 Faculty of Pharmacy, Department of Pharmacy, Al-Zaytoonah University of Jordan, Amman, Jordan; 3 Department of Biopharmaceutics and Clinical Pharmacy, School of Pharmacy, The University of Jordan, Amman, Jordan; 4 School of Pharmacy, Queen’s University Belfast, Belfast, United Kingdom; Jordan University of Science and Technology, JORDAN

## Abstract

**Background:**

Oral contraceptive pills (OCPs) are considered one of the most important birth control methods globally. However, these pills were designed for female administration rather than males. This study was designed to investigate patterns of OCPs use and abuse among Jordanian males, according to the community pharmacists’ observations.

**Method:**

A mixed-method explanatory sequential study was conducted using an online self-administered survey, followed by semi-structured in-depth interviews for registered pharmacists, assistant pharmacists and pharmacy interns. The interviews were utilized using a conceptual framework. Inductive thematic analysis and descriptive/regression analyses were completed using Nvivo and SPSS, respectively.

**Results:**

A total of 158 questionnaire responses and 22 interviews were included in our analysis. Around half (48.4%) of the questionnaire responses confirmed that males could use OCPs for hair growth enhancement, muscle gain and acne treatment 12.7%, 31.7% and 4.4%, respectively. Through the interviews, the participating pharmacists highlighted that males use OCPs mostly for bodybuilding purposes, according to recommendations by their coaches at the gym. The most abused OCPs containing estrogen (Ethinyl estradiol) and progestins (Drospirenone or Levonorgestrel).

**Conclusion:**

This study provided insight into unexpected uses of OCPs by males in Jordan. Community pharmacists have a crucial role in the management of OCPs use and abuse. However, restricted regulations and monitoring must be released and implemented on the community to limit such practices.

## 1. Introduction

The non-therapeutic consumption of medications, in terms of abuse and misuse, is recently on the rise. Medication abuse is defined as the usage of drugs for non-medical purposes [1]. The most common medications which are prone to abuse are products containing opioids, stimulants and laxatives [2–4]. However, the abuse of oral contraceptive pills (OCPs) has not been thoroughly examined yet.

OCPs are one of the most common birth control methods in the United States (US), mainly used by females between the ages of 25–44 years old [5]. There are three types of OCPs: combined estrogen-progesterone, progesterone alone and continuous dosing or extended cycle pills [6]. It was reported that the combined oral contraceptive (COC) pills were the most prescribed compared with the other types [6]. The main indication for these pills is pregnancy control; however, the Food and Drug Association (FDA) labeled additional uses for them such as menstrual period disorders (Dysmenorrhea, amenorrhea, oligomenorrhea), acne treatment, hirsutism, polycystic ovary syndrome and many other uses [7]. OCP’s labeled indications are generally approved for females rather than males. The frequent use of OCPs among males may contribute to adverse physiological effects such as gynecomastia, testicle shrinkage, prostate cancer and diminished libido [8].

In Jordan, it was reported that OCPs are the second most common birth control method [9,10], and OCPs are sold over the counter [9]. Such unsupervised practice could be associated with inappropriate administration of many medications [11], including OCPs, as reported by the Jordanian community pharmacist in a previous study [12]. In that study, pharmacists stated that both genders are prone to OCPs improper usage, such as topical application of OCPs for hair growth enhancement and OCPs were used to give a false negative result for an addictive drug screening test [12]. Therefore, this study aims to investigate the patterns of OCPs use and abuse among Jordanian males, according to the community pharmacists’ observations.

## 2. Method

**Fig 1:**
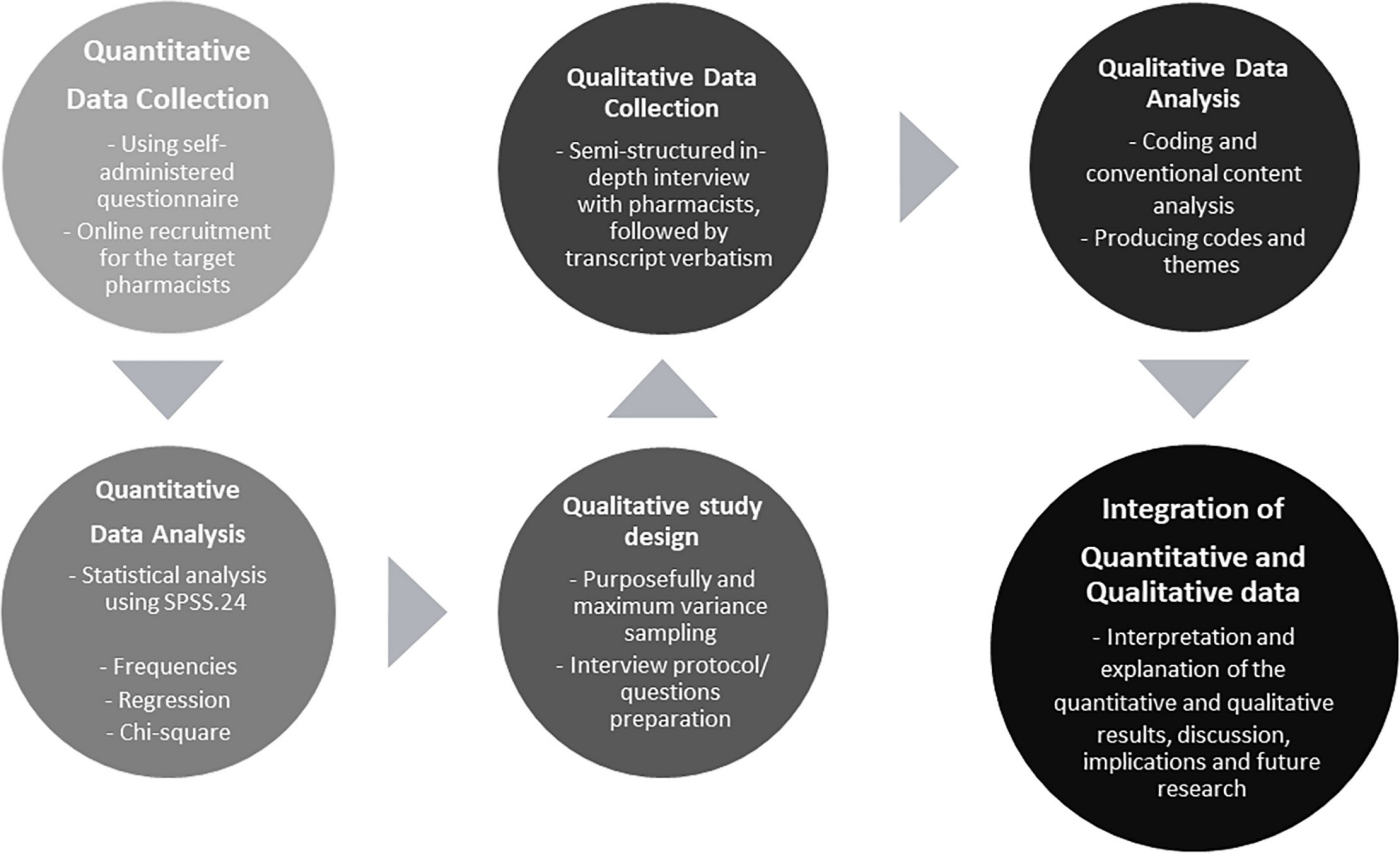
Descriptive scheme for the study mixed sequential method.

A mixed-method explanatory sequential (qualitative and quantitative) study was conducted in order to understand the pharmacist observations about patterns of OCPs use/abuse among Jordanian males. The first phase of the study, which was conducted from March 15^th^ to April 24^th,^ 2020, consisted of a self-administered online survey, targeting Jordanian community pharmacists pharmacy assistants and pharmacy trainees. Pharmacists were recruited through social media platforms (Facebook, WhatsApp, LinkedIn, and Twitter). The second phase of the study, which was carried out after the data analysis of the first phase, from April 30^th^ to May 30^th^, 2020. This phase consisted of online semi-structured in-depth interviews, using Zoom®, with maximum variation purposive sample of community pharmacists. The study was approved by the Institutional Review Board of Applied Science Private University [Ethical approval number: 2019-PHA-14]. The sequential method is represented in Fig 1.

### 2.1 Quantitative method (first phase)

An online self-administered survey has been developed and validated (face and content validity) by clinical researchers to solicit anonymous responses, which were treated confidentially. The survey has been developed using the general principles of good survey design [13]. The surveys contain closed-ended and open-ended questions that are completed within an average of 7 minutes. The survey had been administered in Arabic and English via Google® forms technology. It was explicitly stated for participants that their participation in the study was voluntary and did not pose any risks. Potential participants who completed the survey were considered to have given informed consent for the study participation.

The first draft of the survey was appraised by 20 independent academic staff members who had conducted many surveys in fertility, reproductive health, contraception research in Jordan; also a statistician was consulted at this stage of validation. The final version of the survey was refined according to the provided comments and feedback, translated from English into Arabic and then was back-translated by two specialized academics. The questions were free from medical jargon or difficult terminologies. The final validation stage was the piloting step, which involved 25 academic and 25 non-academic participants. This stage of the study was conducted to enhance clarity, readability, understandability, and confirm the study applicability in the Jordanian community pharmacists. Internal consistency reliability was tested by the Cronbach’s alpha coefficient (= 0.82).

The study survey included three parts. The first part (Part A) comprised nine questions including sociodemographic information; the second part (Part B) consisted of three questions comprising details of knowledge and beliefs of pharmacists about OCPs uses for males. The questions of knowledge were mainly concerned about the presence of possible indications for OCPs in males and the risks of using those pills. The last part (Part C) was focusing on the pharmacist experience and practice towards OCPs usage by males; which consisted of ten multiple-choice questions.

Based on Tabachnick and Fidell recommendation for sample size calculation in analysis, 5–20 subjects per predictor are suggested to be preferable [14]. Based on the number of independent variables levels used in this study (n = 9) and using the number of 10 subjects per predictor level, a minimum sample size of 90 or higher was considered suitable for the purpose of this study.

The eligibility criteria for participation are being Jordanian pharmacists and trainees (who were working/training in community pharmacy). Recruitment was conducted using a convenience sampling method. The inclusion criteria were explained at the start of the survey, which stated: “If you are working or training at a community pharmacy, please let us know if you would like to participate in this survey”.

### 2.2 Statistical analyses

The data of the completed surveys were extracted from an electronic platform and exported to Statistical Package for Social Sciences version 24.0 (SPSS® Inc., Chicago, IL, USA) for the statistical analysis. Descriptive statistics included percentages, means, and frequency distribution, calculated for each question. Descriptive and univariate correlation analyses using Pearson correlation coefficient (r) were used for the correlation, which was conducted at a 5% significance level. A *p*-value of < 0.05 represented a significant difference. Factors affecting the OCPs’ improper uses were analyzed using Univariate and Multivariate logistic regression.

### 2.3 Qualitative method (second phase)

The qualitative study was conducted using semi-structured in-depth interviews for community pharmacists. The sample was recruited purposively then maximum variation sampling was conducted, according to age, gender, workplaces, educational degrees, to reach sample saturation (22 community pharmacists). Purposeful sampling directed the researcher to find community pharmacists with pure experiences regarding OCPs usage among males. The subjects from the first quantitative responders were involved in the second part of the study. In this regard, introductory pilot interviews were employed to find the pharmacists who provide fruitful and detailed responses then the selected pharmacists participated in in-depth interviews.

A series of questions regarding the use of OCPs among males were asked and answered freely by the participants. This research instrument was reviewed by a group of leading clinical pharmacy researchers to ensure the suitability of the measurement tool. The interview included three probing questions. The first was “From your experience, please describe the most common patterns for the OCPs use among males”, and the second was “what are the most common abused OCPs among males?”, and finally “What is the best way to limit the abuse of OCPs among males?”, each interview lasted for 25 minutes. The participants were informed that all the data will be treated confidentially and signed electronic informed consents. The interviews were recorded and transcribed verbatim. Transcripts were written in Arabic (the native language of Jordan), translated into English, checked for clarity and accuracy, uploaded to NVivo12 (qualitative data analysis software; QSR International Pty Ltd. Version 12) for coding and then analyzed using inductive thematic analysis using conceptual framework methods [15]. The inter-rater reliability was assessed using Cohen’s kappa. The kappa value for the three coders was 0.85, indicating good agreement between the coders. According to Connelly’s published study, different researchers independently re-checked the trustworthiness and the accuracy of transcription, translation and inter-coder reliability [16].

## 3. Results

### 3.1 Sociodemographic characteristics of the participants in both quantitative and qualitative phases

In this study, the recruited sample was 162 community pharmacists who met the inclusion criteria. Out of the total 162 completed questionnaires (response rate = 98%), four forms (2.4%) were excluded from the study due to incomplete responses, accordingly, 158 (97.5%) questionnaires were included in the analysis. The majority of the participating pharmacists were females (n = 118, 74.2%) and more than half of them (n = 94, 59.5%) were <40 years old and more than two-third were holding bachelor’s degrees or postgraduates (n = 111, 70.2%). About half of the pharmacists (n = 75, 47.5%) had been working in community pharmacies for more than 10 years, which is located in the capital of Jordan "Amman" (n = 115, 72.3%). Around 74% (n = 117) of the pharmacy’s customers were classified as middle social class, from the pharmacists’ perception. It was noteworthy that 44.7% (n = 71) of the pharmacies were adjacent to sports gyms (Table 1).

**Table 1:** Sociodemographic characteristics of the participants in both quantitative (n = 158) and qualitative phases (n = 22).

Characteristic	Questionnaire Sample n (%)	Interviews Sample n (%)
**Gender**		
• Female	118 (74.2)	12 (54.5)
• Male	41 (25.8)	10 (45.5)
**Age (years)**		
• 21–30	35 (22.2)	5 (22.7)
• 31–40	59 (37.3)	5 (22.7)
• 41–50	54 (34.1)	7 (31.8)
• >50	10 (6.3)	5 (22.7)
**Education**		
• Diploma	12 (7.5)	0
• Postgraduate degree	11 (6.9)	1 (4.5)
• Pharmacy student +trained in a pharmacy	35 (22.2)	4 (18.2)
• Bachelor’s degree	100 (63.3)	17 (77.3)
**Years of experience**		
• <5	35 (22.2)	4 (18.2)
• 5 to 10	48 (30.4)	4 (18.2)
• 11 to 15	58 (36.7)	5 (22.7)
• 16–20	8 (5.0)	5 (22.7)
• >20	9 (5.7)	4 (18.2)
**Position in the pharmacy**		
• Employee pharmacist	94 (59.5)	9 (40.9)
• Trainee in a pharmacy	37 (23.4)	6 (27.3)
• Pharmacy owner	27 (17.6)	7 (31.8)
**Province where you work**		
• The capital of Jordan (Amman)	115 (72.3)	13 (59.1)
• East of Jordan	9 (5.7)	2 (9.1)
• West of Jordan	6 (3.8)	3 (13.6)
• North of Jordan	16 (10.1)	2 (9.1)
• South of Jordan	1 (0.6)	2 (9.1)
**The most common social class distribution of the pharmacy customers**		
• Low	21 (13.3)	5 (22.7)
• Middle	117 (74.0)	13 (59.1)
• High	20 (12.7)	4 (18.2)
**The presence of sport gym around the pharmacy**		
• Yes	71 (44.7)	14 (63.6)
• No	63 (39.6)	8 (36.4)
• Not sure	24 (15.1)	0

The sociodemographic data of the qualitative phase was representing the maximum variation sample. A total of 22 pharmacists were involved in the study. Females percentage was slightly predominant over males (n = 12, 54.5%), less than one third were 41–55 years old (n = 7, 31.8%) and (n = 17, 77.3%) were holding a bachelor’s degree (Table 1).

### 3.2 Knowledge and beliefs of pharmacists about male’s usage of OCPs

The majority of the participants (n = 77, 48.4%) agreed that OCPs could be used by males. The pharmacists reported that males could use OCPs for muscle gain purposes, enhancement of hair growth and acne treatment in 31.7%, 12.7% and 4.4%, respectively. Pharmacists’ knowledge about possible OCPs side effects, when used by males, was assessed. The most reported side effects were gynecomastia, decreased libido, mood changes, and increased body weight (69.8%, 64.8% 64.8%, 57.9%) respectively (Table 2).

**Table 2:** Pharmacists knowledge and beliefs about the possible uses of OCPs among males (n = 158).

Question	n	%
**According to your knowledge and opinion, could males use OCPs?**		
• Yes	77	48.4
• No	52	32.7
• Not sure	29	18.2
**According to your knowledge and opinion, what are the possible uses of OCPs for males?**		
• Enhancement of hair growth	20	12.7
• Muscle gain (bodybuilding)	50	31.7
• Acne Treatment	7	4.4
• No uses	52	32.7
• Other uses	3	1.9
• Not sure	26	16.5
**Which of the following could be possible side effects of OCPs, if used by males? (Yes/No)** *		
• Acne flare-up	64	40.3
• Altered libido	103	64.8
• Night sweats	61	38.4
• Mood changes	103	64.8
• Gynecomastia	111	69.8
• Suppression of high lipoprotein cholesterol (HDL)	48	30.2
• Reversible reduction in testicular volume	64	40.3
• Increased body weight	92	57.9

• The showed results represent the “Yes” answers.

### 3.3 Pharmacist experience and practice towards OCPs usage by males

Sixty-two percent of the participating pharmacists acknowledged that they dispense OCPs without prescription, and around 58% of them were dispensing OCPs for males for their personal use. In particular, for muscle gain (36.5%) and hair growth enhancement (20.8%). Some of the participating pharmacists also indicated that most males who used to use OCPs were non-familiar customers (n = 43, 27.7%) and most commonly aged 20–35 (n = 114, 71.7%). Study participants stated that the males’ OCPs users either directly ask and admit their need for these pills or are recognized by their facial expression and body language, 21.5% and 27.2%, respectively (Table 3).

**Table 3:** Pharmacist experience and practice towards OCPs usage by males (n = 158).

Question	n	%
**Have you ever dispensed OCP s in your pharmacy to males (for their personal use)**		
• Yes	92	57.9
• No	34	21.4
• I have never asked about the user	32	20.8
**Have you ever dispensed OCPs to males for the following uses?**		
• Enhancement for the hair growth	32	20.8
• Muscle gain (bodybuilding)	58	36.5
• Acne treatment	2	1.3
• Never	66	41.5
**The most common ages of males came to the pharmacy asking for OCPs (years)**		
• < 20	12	8.2
• 20–35	114	71.7
• 36–50	30	18.9
• > 50	2	1.3
**Have you ever noticed any inappropriate use of OCPs by males?**		
• Yes	68	43.4
• No	44	27.7
• Not sure	46	28.9
**What was the kind of OCPs’ male customers in the last 3–6 months**		
• Known customers	3	1.9
• Strangers	43	27.0
• Known and strangers	28	17.6
• Not sure	84	52.8
**How many males came to your pharmacy asking for OCPs in the last 3–6 months**		
• <5	120	75.9
• >5	38	24.1
**How could you recognize the males who use OCPs inappropriately**		
• From the facial impressions and body language	43	27.2
• They admit their needs directly	34	21.5
• Hard to recognize	60	38.0
• All the options	21	13.3

Multivariate logistic regression results exhibited a significant positive correlation *(p*< 0.05) between the pharmacist exposure to males OCPs abuse cases and the following variables: age of the pharmacist, female gender, the length of pharmacist experience (in years), the relatively low social class distribution of the pharmacy customers, Table 4.

**Table 4:** Summary of the logistic regression analysis (Univariate and Multivariate) to assess predictors associated with the exposure of participating pharmacists to OCPs use among males.

Independent factors	Univariate		Multivariate	
	Beta	*p*-value	Beta	*p*-value
**Age**				
• ≤40 years	Reference			
• >40 years	0.512	**0.033**	0.499	**0.047**
**Gender**				
• Female	Reference			
• Male	-0.143	**0.001**	-0.128	**<0.001**
**Education**				
• ≥ University degree	Reference			
• < University degree	0.711	**0.127**	-------	-------
**Years of experience**				
• ≤10 years	Reference			
• >10 years	0.349	**0.003**	0.325	**<0.001**
**Province of the work**				
• The capital (Amman)	Reference			
• Outside the capital	-0.473	0.275	-------	-------
**Social class distribution of the pharmacy customers**				
• Low	Reference			
• Moderate/high	-0.067	**0.005**	-0.063	**<0.001**
**Presence of Sport Gym around the pharmacy**				
• Yes	Reference			
• No	-0.352	**0.052**	-------	-------

Significance (*p*<0.05) presented in bold numbers.

### 3.4 Qualitative phases results

#### Theme 1: The most common patterns of OCPs abuse among males

Pharmacists confirmed that most of the male OCPs users were 22–35 years old. The majority of the participants highlighted that most of the males use OCPs for bodybuilding purposes, according to recommendations by their coaches at the gym. They also reported that the presence of a gym facility around the pharmacy increases the potential to observe such cases. All male customers were asking for OCPs containing estrogen (Ethinyl estradiol) and progestins (Drospirenone or Levonorgestrel). Participant 4 declared that “*After a period of 3 months*, *the same customers came to the pharmacy asking for Tamoxifen (selective estrogen receptor modulator) or Clomiphene citrate tablets (nonsteroidal*, *ovulatory stimulant)*, *claiming that this will clear their bodies from the side effects of OCPs*”. All the participants confirmed that there is general use for hormonal products among gyms, including OCPs and testosterone.

Thirty percent of the participating pharmacists said some of the customers came to their pharmacy asking for OCPs for drug-addiction-related issues. Participant 5 stated, “*most commonly*, *cocaine abusers came to ask for OCPs approximately five hours before their annual urine test of illicit drugs in order to give a negative result”*. Participant 7 also mentioned that “*some customers usually asking for oral contraceptives to mask the results of their urine test and for eye drops containing Naphazoline HCl and Chlorpheniramine Maleate to manage their eye symptoms related to addiction”*.

On the other hand, the pharmacists indicated that males use OCPs for hair growth purposes. Participant 10 said, “*As the men’s beauty centers recommend males to grind OCPs and mix them with shampoo for topical application to improve their hair growth”*. Participant 13 acknowledged, “*Such practice is more common in the presence of beauty centers around the pharmacy”*. As well, fifty percent of the participants stated that beauty centers also recommend topical application of OCPs (grinded pills mixed with topical products) for acne treatment.

#### Theme 2: The role of pharmacist and pharmacy policymakers in the management of OCPs abuse among males

Most of the participants said that they could distinguish the abusers of OCPs by their facial impressions and body language/shape, mainly for addictive customers who have certain physical signs that enable the pharmacist to suspect the abuse. For example, looking unkempt, bloodshot eyes, slurred speech and unusual pupil size. As well as frequent visits to the pharmacy asking for the same products and even sometimes the customer explicitly clarify his need. Additionally, all participants agreed on the importance of the pharmacist role to fight the abuse of OCPs and came up with some recommendations such as “*Once I know that there is any misuse for OCPs*, *I would advise the customer to avoid this practice and mention the side effects (especially the risk for impotence) for him*,” said participant 20. In addition, they advocated the conduction of awareness campaigns, videos and distribution of pamphlets to increase knowledge and awareness about the proper use of OCPs, especially for gyms and beauty centers. Finally, the participant emphasized that pharmacists should report the abuse or misuse cases to the policymakers such as the Jordanian Pharmacist Association (JPA) and pharmacovigilance committee in the Jordan Food and drug association (JFDA) to monitor such practice.

## 4. Discussion

The abuse of body performance- or appearance- enhancing drugs by males has been increasing all over the world [17,18], as well as in the Middle Eastern countries [18], which mostly include androgens [18,19] and growth hormones [20]. However, the abuse of OCPs for these aforementioned purposes has never been reported in the literature. In the current study, community registered pharmacists, pharmacy assistants and interns were able to identify and report unanticipated patterns of OCPs use among Jordanian males, which provides an insight into the pharmacists’ role in patient education to limit medication abuse.

Half of the pharmacists participating in this study were aware of the possibility of OCPs use by males for body appearance enhancing purposes such as muscle augmenting, hair growth as well as a treatment for acne. They were also knowledgeable about the side effects of OCPS in males, such as gynecomastia, mood changes, altered libido, and weight gain. These side effects were actually similar to those reported in a recent study that evaluated hormonal contraception for men [21]. Most males who are buying OCPs were young (25–35 years old), and this was comparable to other studies that evaluated the abuse of androgenic steroids by male athletes in Jordan, which found that the mean age of the abusers was 28.1 years [22]. Tahtamouni [22] showed that in 42.9% of the abuse cases, gym coaches were responsible for recommending medication use/abuse. Similar to our study, participants also confirmed that the presence of a gym facility around the pharmacy increases the potential to observe such cases. Similarly, in a neighboring country, the Kingdom of Saudi Arabia, a survey in 4860 male participants showed that androgens [23] were highly abused in gyms and 6.1% abused OCPs for breast enlargement and used it just before exercise. This abuse was correlated significantly with the duration of gym participation [24]. The accessibility and affordability of OCPs make them more prone to misuse/abuse [25].

A previous Jordanian study has demonstrated that around forty percent of the pharmacists used to refuse OCPs dispensing in cases of suspected improper use, yet the rest of them would agree to do so [12]. Unfortunately, community pharmacists in Jordan still have dispensed OCPs without prescription, which is contrary to the national regulations by the JPA or JFDA [26]. The lack of control over OCPs prescribing will lead to the augmentation of the problem of improper OCPs use. Previous studies showed that these practices can be attributed to the pressure on the community pharmacists to sell the medication due to financial considerations of the pharmacy owners, or the continuous push from the pharmaceutical companies [27]. This issue is very important at the national as well as the international level, hence, many studies have investigated the possibility of changing the status of OCPs to be an over the counter medication and expanding the scope of pharmacists’ practice beyond counseling and education to prescribing [28–31]. This suggests that there is a need for strict regulations and guidance to control any possibility of improper medication use. Still, our study showed that those pharmacists who refused to dispense emphasized that pharmacists should report the abuse or misuse cases to the policymakers such as the JPA and pharmacovigilance committee in the JFDA to monitor such practice.

Remarkably, most of the documented cases of the improper use of OCPs in this study have been rarely recognized in previous studies. Our qualitative analysis showed that OCPs were abused to mask the results of addiction to drug urine tests. It was shown that several adulterants could be added to urine in order to give a false-negative drug test, which could deter the ability to monitor illicit drug use [32]. These can include oxidizing chemicals, such as nitrite or peroxide, as well as non-oxidizing chemicals. Hajhashemi et al. (2007) conducted an in vitro and in vivo study assessing the interaction of OCPs (ethinylestradiol, levonorgestrel (LN), and both of them) at a high dose with a urine morphine diagnostic test, after reporting plenty of claims about this issue [33]. The results of that study confirmed the absence of such an interaction, which strongly suggests there is a need to stop misusing these medications. However, such practices are still present according to our study findings, which should highlight the need for further future studies to better understand this issue and devise suitable recommendations for policymakers.

A few participants reported the use of clomiphene to manage the side effects of OCPs. While this was never reported in the literature, one case report showed that a young man admitted suffering from severe depression and libido due to steroid abuse and was using clomiphene to alleviate these adverse effects [34]. Clomiphene is used in females to induce ovulation, and also has an off-label use for the treatment of hypogonadism [35,36]. It has both estrogenic and antiestrogenic properties and initiates a series of endocrinologic events that eventually lead increase in steroidogenesis [36]. Still, the mechanism by which clomiphene can alleviate the adverse effects of OCPs is yet unclear.

Regarding bodybuilding and muscle gain the evidence for the beneficial effect of OCPs is relatively weak. However, a study conducted by Dalgaard et.al (2019) has revealed the positive influence of OCPs on the adaptations to resistance sports training [37]. As, they disclosed that OCPs have a synergistic effect with the regular training on the activation of skeletal muscle signaling through the involvement of estrogen receptors [37,38]. This effect was evident with the ethinylestradiol-containing pills and affect only type l body muscle-fibers, but the overall muscle strength was not influenced significantly [37]. That study was conducted over females who practiced the resistant training. Regarding males, studies have shown that estrogen receptors (E2) found in muscles do not appear to be important for muscle size or strength [39,40]. Accordingly, estrogen-containing OCPs are not supposed to have a beneficial effect for bodybuilding purposes, thus contradicts the study findings. Instead, OCPs could have substantial side effects such as breast tenderness and decreased libido [41]. Accordingly, educating pharmacists about the latter will prompt them to discourage men from abusing OCPs for such purposes. Advanced awareness campaigns about the harmful effects of OCPs are required, especially, for males who are concerned about the muscle gain using these medications. Besides, close monitoring and serious action are required for the possible sources for such practices such as gyms coaches.

In this study, the reporting of OCPs abuse cases by males was correlated to many factors according to our study results, including the extent of the pharmacist’s experience. Usually, the experience in the pharmacy profession may strengthen the potential of the pharmacist to distinguish the cases of proper/abuse use of medication [42]. Moreover, the low social class distribution of pharmacy customers was one of the significantly affecting factors. The effect of socio-economic distribution on substance use (e.g. illicit drugs and alcohol) was investigated in the literature, not about OCPs, which reveals that “low social status report more environmental challenges and less psychosocial resources and that this can lead to feelings of hopelessness and a loss of coping ability” [43]. This could explain the abuse of OCPs by males to mask the urine test results of the illicit drug.

In Jordan, the policymakers and stakeholders (JFDA) emphasized that birth control pills should be handled under prescription, and till the time of writing this study it is not allowed to handle such medication as over-the-counter medications [44]. Nevertheless, a lack of surveillance and strict control opens the chance to abuse the OCPs and make them accessible to the abusers, which could ultimately lead to an increase of side effects and complications [45]. However, there is limited documentation on cases of the use and abuse cases of OCPs among males.

## 5. Strengths and limitations

This has been the first study in Jordan to addresses various patterns of use/abuse of OCPs by males, which are encountered in the community pharmacy setting. However, several limitations have been raised. The first limitation of this study was the survey was conducted online due to the COVID-19 pandemic that started around January of 2020 [46], along with the lockdown which was enforced by the law (till mid-June 2020). Another limitation was the use of convenient non-random sampling. Also, the period of data collection for the survey was short (March 15th to April 24th) with a relatively small sample size. A future study is required for a longer time, more responses, from wider geographic locations for more generalizable findings. Finally, limitations in the qualitative strand were faced during online interviews (Internet connection, inability to fully observe body language as the camera only shows the face.

## 6. Conclusion

This study provided insight into unexpected uses of OCPs by males in Jordan. Community pharmacists have a crucial role in the management of OCPs use and abuse, However, restricted regulations and monitoring must be released and implemented on the community to limit such practices. These regulations should be highlighted by both policymakers and drug regulatory institutions in Jordan. Future studies should seek a larger sample size and diverse geographical locations. In addition, there is a deep need for national educational and awareness programs for the Jordanian community about the safe and proper use of OCPs.
